# Supplementary material for: Exploring attitudes and acceptance of artificial intelligence in multiple sclerosis from the patient perspective
Source: PLOS Digit Health. 2026 Jul 1;5(7):e0001236. doi: 10.1371/journal.pdig.0001236 (PMC13322512; doi:10.1371/journal.pdig.0001236)
Supplement: S1 Table — (DOCX) [file pdig.0001236.s001.docx]

**S1 Table. Survey instrument (English translation)**

| Domain | Item ID | English Translation of Question | Response Options |
| --- | --- | --- | --- |
| 1. Participant Characteristics | P1 | How old are you? | Numeric (Range: 18–99 years) |
|  | P2 | Which gender do you identify with? | Single Choice: Female, Male, Diverse |
|  | P3 | Please state the first three digits of your postal code. | Numeric |
|  | P4 | When were you diagnosed with MS? | Numeric (Year, Range: 1960–2025) |
|  | P5 | What is your diagnosed MS course? | Single Choice: Relapsing-Remitting MS (RRMS), Primary Progressive MS (PPMS), Secondary Progressive MS (SPMS) |
|  | P6 | What is your highest educational degree? | Single Choice: nN school leaving certificate, basic secondary school leaving certificate, intermediate secondary school leaving certificate, university entrance qualification (Abitur), completed vocational training, University of Applied Sciences degree, and university degree |
|  | P7 | Which of the following descriptions best describes your degree of disability? (PDDS 0-8) | 0) Normal: I may have mild symptoms, mostly sensory due to MS, but they do not limit my activity. If I have a relapse, I return to a normal state after the relapse.  1) Mild Disability: I have noticeable symptoms of MS, but they are minor and have only a small influence on my lifestyle.  2) Moderate Disability: I have no limitations walking, but due to MS I have significant problems that limit my daily activities in other ways.  3) Walking Disability: MS impairs my activities, especially walking. I can work a full day, but physically strenuous activities are more difficult. During a relapse, I might need some support, but usually no cane.  4) Occasional use of a walking aid: I use a cane or other support (e.g., touching walls) to walk, always or partially, especially outside. I think I can walk 7.5 meters in 20 seconds without a cane, but need support for longer distances (e.g., 300 meters).  5) Permanent use of a walking aid: I need a cane or crutch for 7.5 meters and use furniture or walls for support in the house. For longer distances, I might use a wheelchair or scooter.  6) Bilateral support: To walk 7.5 meters, I need two canes or a walker and might use a wheelchair or scooter for longer distances.  7) Wheelchair/Scooter: My main means of transport is the wheelchair. I might be able to stand or take a few steps, but I cannot walk 7.5 meters, even with crutches.  8) Bedridden: Cannot sit in a wheelchair for longer than one hour. |
| 2. Technology and AI access and use | T1 | Which of the following devices do you use? | Multiple Choice: Computer / PC, Laptop/Notebook, Smartphone/Tablet, Wearables (e.g., Smartwatch), Mobile phone without internet access |
|  | T2 | How often do you normally use a Computer/PC or a Laptop/Notebook? | Single Choice: Multiple times a day, Once a day, Multiple times a week, Once a week, Rarely/Never |
|  | T3 | How often do you use a Smartphone/Tablet? | Single Choice: Multiple times a day, Once a day, Multiple times a week, Once a week, Rarely/Never |
|  | T4 | What do you regularly use your Computer/PC or Laptop/Notebook for? | Multiple Choice (Selected Options): Word processing, E-mails, Entertainment, Seeking information about MS |
|  | T5 | What do you use the Smartphone/Tablet for? | Multiple Choice (Selected Options): Making calls, Text messages, Audio/Video messages, Entertainment, E-mails, Appointment scheduling, Seeking information about MS |
|  | T6 | How often do you normally surf the internet? | Single Choice: Multiple times a day, Once a day, Multiple times a week, Once a week, Rarely/Never |
|  | T7 | What do you already know about Artificial Intelligence (AI)? | Single Choice: I have no knowledge (never heard of it), I have little knowledge, I have good knowledge, I am an expert |
|  | T8 | How often do you use AI (e.g., chatbots)? | Single Choice: Daily, Multiple times a week, Once a week, Less often, Never |
|  | T9 | How often do you use AI (e.g., chatbots) for health-related questions? | Single Choice: Daily, Multiple times a week, Once a week, Less often, Never |
| General Attitudes toward AI in MS Care | A1 | What potential do AI applications have for MS treatment/care? | 5-Point Likert: 1=Very Pessimistic to 5=Very Optimistic |
|  | A2 | “AI should increasingly be used in MS care” | 5-Point Likert: 1=Strongly Disagree to 5=Strongly Agree |
|  | A3a | How comfortable do you feel using AI for: Early detection of possible neurological symptoms (e.g., via fitness trackers, text entry)? | 5-Point Likert: 1=Not at all comfortable to 5=Very comfortable |
|  | A3b | How comfortable do you feel using AI for: Disease diagnosis? | 5-Point Likert: 1=Not at all comfortable to 5=Very comfortable |
|  | A3c | How comfortable do you feel using AI for: Treatment selection? | 5-Point Likert: 1=Not at all comfortable to 5=Very comfortable |
|  | A3d | How comfortable do you feel using AI for: Management of chronic symptoms (e.g., fatigue & mobility)? | 5-Point Likert: 1=Not at all comfortable to 5=Very comfortable |
|  | A4 | Assuming an AI had about the same accuracy in diagnostics as physicians. What would you prefer? | Single Choice: Physicians alone. / Joint decision-making with final call by physician. / Joint equal say. / Final call by AI. |
|  | A5 | I would prefer to visit MS centers that utilize suitable AI applications as part of the treatment. | 5-Point Likert: 1=Strongly Disagree to 5=Strongly Agree |
|  | A6 | I would like an AI tool developed specifically for MS that I could consult in addition to my physician. | 5-Point Likert: 1=Strongly Disagree to 5=Strongly Agree |
