# Supplementary material for: Exploring attitudes and acceptance of artificial intelligence in multiple sclerosis from the patient perspective
Source: PLOS Digit Health. 2026 Jul 1;5(7):e0001236. doi: 10.1371/journal.pdig.0001236 (PMC13322512; doi:10.1371/journal.pdig.0001236)
Supplement: S2 Table — (DOCX) [file pdig.0001236.s002.docx]

**S2 Table. Univariable associations with AI attitudes composite in people with Multiple Sclerosis**

| **Predictor** | **B** | **95% CI for B** | **β (std.)** | **p** | **N** |
| --- | --- | --- | --- | --- | --- |
| Education (High vs Low) | 0.09 | −0.11 to 0.28 | 0.06 | 0.397 | 241 |
| Age (years) | −0.01 | −0.02 to −0.00 | −0.17 | 0.011 | 239 |
| Gender (Male vs Female) | 0.20 | −0.02 to 0.41 | 0.12 | 0.072 | 238 |
| Region (West vs East) | 0.34 | 0.15 to 0.54 | 0.23 | <0.001 | 207 |
| Disease duration (years) | −0.00 | −0.01 to 0.01 | −0.05 | 0.444 | 239 |
| Disability (PDDS) | −0.07 | −0.11 to −0.02 | −0.18 | 0.004 | 241 |
| AI knowledge (High vs Low) | 0.41 | 0.22 to 0.60 | 0.26 | <0.001 | 241 |
| General AI use (Frequent ≥1×/week vs Infrequent <1×/week) | 0.67 | 0.47 to 0.86 | 0.40 | <0.001 | 241 |
| Health-related AI use (Frequent vs Infrequent) | 0.26 | −0.05 to 0.58 | 0.10 | 0.104 | 241 |

Results from separate univariable linear regression analyses with the AI attitudes composite score as the dependent variable. B: unstandardized regression coefficient; 95%CI: 95% confidence interval; β (std.): standardized regression coefficient; N: number of valid cases included in the analysis; PDDS: Patient Determined Disease Steps. Reference categories for categorical variables are: Education (Low), Gender (Female), Region (East), AI knowledge (Low), General AI use (Infrequent), and Health-related AI use (Infrequent).
