# Supplementary material for: Exploring attitudes and acceptance of artificial intelligence in multiple sclerosis from the patient perspective
Source: PLOS Digit Health. 2026 Jul 1;5(7):e0001236. doi: 10.1371/journal.pdig.0001236 (PMC13322512; doi:10.1371/journal.pdig.0001236)
Supplement: S3 Table — (DOCX) [file pdig.0001236.s003.docx]

**S3 Table. Missingness and analytic denominators across key participant characteristics, predictors, and major outcome items in people with Multiple Sclerosis**

| **Domain** | **Variable** | **Valid n** | **Missing n (%)** |
| --- | --- | --- | --- |
| Participant characteristics | Age | 239 | 2 (0.8) |
|  | Gender | 238 | 3 (1.2) |
|  | Postal code information | 238 | 3 (1.2) |
|  | Region (East vs West) | 207 | 34 (14.1) |
|  | Year of diagnosis | 239 | 2 (0.8) |
|  | Disease duration | 239 | 2 (0.8) |
|  | MS course | 241 | 0 (0.0) |
|  | Education | 241 | 0 (0.0) |
|  | Disability level (PDDS) | 241 | 0 (0.0) |
| Key predictors used in regression models | AI knowledge group | 241 | 0 (0.0) |
|  | General AI use group | 241 | 0 (0.0) |
|  | Health-related AI use group | 241 | 0 (0.0) |
| Major outcome items | AI potential | 241 | 0 (0.0) |
|  | AI should increasingly be used in MS care | 232 | 9 (3.7) |
|  | Comfort with AI for symptom detection | 241 | 0 (0.0) |
|  | Comfort with AI for diagnosis | 241 | 0 (0.0) |
|  | Comfort with AI for treatment selection | 241 | 0 (0.0) |
|  | Comfort with AI for chronic management | 241 | 0 (0.0) |
|  | Preference for AI-enabled MS centers | 241 | 0 (0.0) |
|  | Desire for an MS-specific AI tool | 241 | 0 (0.0) |
|  | AI versus physician preference | 241 | 0 (0.0) |
|  | AI attitudes composite score | 241 | 0 (0.0) |

Missingness overview for key variables included in the main analyses. Missing data were handled using complete-case analysis for each model. Region-based analyses were restricted to participants with valid region information (n = 207). The primary multivariable linear regression model included n = 204 participants. AI: Artificial Intelligence; MS: Multiple Sclerosis; PDDS: Patient-Determined Disease Steps.
