# Supplementary material for: Exploring attitudes and acceptance of artificial intelligence in multiple sclerosis from the patient perspective
Source: PLOS Digit Health. 2026 Jul 1;5(7):e0001236. doi: 10.1371/journal.pdig.0001236 (PMC13322512; doi:10.1371/journal.pdig.0001236)
Supplement: Attachment — Submitted filename: Response to Reviewers_PLOS_[PDIG-D-26-00145].pdf [file pdig.0001236.s011.pdf]

Po-Chih Kuo, Ph. D.

Section Editor

PLOS Digital Health

11th April, 2026

Dear Dr. Kuo,

we thank you and the reviewers for the feedback regarding our manuscript NRAP-D-23-00095 entitled "Exploring Attitudes and Acceptance of Artificial Intelligence in Multiple Sclerosis from the Patient Perspective".

You can find a point-by-point response to the second round of the review report from the reviewers.

Reviewer #1:

"Ma-1) Recruitment strategy and selection bias

Concern: Recruitment through two academic MS centers and multiple digital channels (social media/newsletter/podcast/website) strongly selects for digitally engaged respondents and may create systematic differences by recruitment pathway. The observed East/West differences in AI acceptance may reflect recruitment mix and center catchment rather than true regional variation.

Requested revision:

•Report recruitment sources quantitatively (e.g., center A vs center B; clinic vs online; specific channels if available).

•Compare key baseline characteristics and AI familiarity/usage by recruitment source.

•Re-estimate the "region" association adjusting for recruitment channel/center, or provide sensitivity analyses stratified by recruitment source.

•Temper interpretation of East/West differences unless robustness is demonstrated."

Thank you very much for pointing this out. We agree that recruitment strategy may have influenced the composition of the sample and thus the observed regional differences. The survey was intentionally implemented as an anonymous, low-barrier, web-based questionnaire with decentralised dissemination across clinical and digital channels. Because no participant-level source-tracking variables were collected, individual responses could not be assigned to a specific recruitment route or recruiting centre after data collection. Unfortunately, this prevented quantitative comparisons by recruitment source and precluded centre-adjusted or source-stratified sensitivity analyses.

To address this as transparently as possible, we have now clarified the recruitment procedures in the Methods, strengthened the limitation statement in the Discussion, and added explicit reporting of missingness and analytic denominators. All region-based analyses were restricted to valid cases. However, as you mentioned, in the absence of participant-level recruitment-source data, we cannot exclude that the observed East/West difference partly reflects differences in recruitment mix, centre catchment, or other unmeasured contextual factors rather than a robust regional effect. We have therefore tempered the interpretation of these results and revised the manuscript to explicitly acknowledge that the observed East/West association may reflect, at least in part, differences in recruitment composition or centre catchment rather than true regional variation.

"Ma-2) Missing data and denominators need systematic reporting and consistent handling

Concern: The supplementary material indicates substantial missingness for some variables (e.g., region responses not available for all participants). Variable denominators differ across analyses; however, the main manuscript does not provide a clear missingness overview or a consistent analytic approach.

Requested revision:

•Add a missingness table (per variable and per key item/domain) and explicitly state how missing data were handled in each model.

•Ensure all tables/figures clearly state denominators (N) for each item/domain and for subgroup comparisons."

We agree and have addressed this directly. We added a new supplementary table reporting missingness for all key participant characteristics, predictors, and major outcome items. We also revised the Methods to state explicitly that missing data were handled using complete-case analysis for each model. In addition, we now report valid denominators for all key item-level outcomes and subgroup comparisons, and we state the analytic sample size for the revised primary multivariable model.

"Ma-3) Outcome processing and dichotomization risk information loss

Concern: Several predictors are dichotomized (e.g., median splits for age/disease duration; categorical collapses for Likert outcomes). While the supplementary clarifies how thresholds were chosen, these decisions still reduce information and may attenuate or distort associations.

Requested revision:

•Consider continuous modeling for age and disease duration (with non-linearity checks if needed).

•Provide sensitivity analyses using the full 5-level ordinal outcomes (or justify the collapsing empirically)."

We agree that preserving information is important. For the primary multivariable regression model, age and disease duration were already entered as continuous predictors, and to address the reviewer's concern more directly we additionally tested whether nonlinearity improved model fit by adding quadratic terms for both variables. These analyses did not indicate meaningful nonlinearity. To facilitate a direct and transparent comparison, we plotted the new sensitivity analyses for the secondary ordinal models using the full 5-level Likert outcomes rather than the collapsed 3-level outcomes. These yielded similar conclusions: frequent general AI use remained the most consistent predictor of higher acceptance, regional associations remained present across several domains, and older age remained negatively associated with AI-supported chronic management. We have therefore retained the collapsed 3-level presentation in the main manuscript for interpretability, while adding a justification in the methods section and the full-scale sensitivity analyses to the supplement.

"Ma-4) Reverse causality and self-selection

Concern: The observed association between frequent AI use and greater acceptance is plausible but cross-sectional. Reverse causality is equally plausible (those favorable to AI are more likely to use it). Survey participation itself may also be correlated with positive attitudes toward digital tools.

Requested revision:

•Replace causal language ("driven by," "key lever") with associative phrasing.

•Expand limitations to explicitly discuss reverse causality and self-selection.

We have revised the manuscript throughout to avoid causal overinterpretation. We now consistently describe frequent general AI use as an associate/correlate of acceptance rather

than a driver. We also expanded the Limitations to note that reverse causality is plausible, meaning that respondents who were already more favorable toward AI may have been more likely both to use AI frequently and to participate in this survey. This issue is especially relevant in a digitally distributed, cross-sectional survey such as ours.

Ma-5) Internal numerical inconsistency in Table 2

Concern: Table 2 contains at least one implausible estimate: "Health-related AI use" shows B=0.070 with a 95% CI 0.251 to 0.391, which cannot be correct (CI does not include the point estimate and is far from it). This undermines confidence in the regression output and needs correction.

Requested revision:

•Audit all coefficients, confidence intervals, and denominators across manuscript and supplement; ensure tables reproduce the model outputs exactly."

We thank the reviewer for identifying this typo (a "minus sign" missing). We audited all coefficients and confidence intervals in the primary regression table and corrected the inconsistency.

"Ma-6) Survey reporting completeness: consider using the CROSS checklist

Concern: Several reporting gaps (recruitment sources/denominators, missingness, questionnaire development and validation steps) are common in survey research and can be addressed systematically.

Requested revision:

•I recommend reporting the study in line with the CROSS (Consensus-Based Checklist for Reporting of Survey Studies), either by adding a completed checklist as supplementary material and/or explicitly mapping key items (sampling frame, recruitment, response rates, instrument development, missing data handling) in the Methods and Supplement. This would improve transparency and facilitate reproducibility."

We agree and have added a completed CROSS checklist as supplementary material. We also revised the Methods and Supplement to more explicitly describe the sampling frame, recruitment strategy, instrument development, and missing-data handling.

"1.Consider adjusting (or at least describing) potential center effects, given recruitment from two academic centers."

We agree that potential center effects are relevant. However, because recruiting center was not captured at participant level, such analyses were not possible. We now state this explicitly and list it as a limitation.

"2.Minor language/formatting edits (typos, spacing, consistent terminology) to improve readability.

We carefully revised the manuscript for style, spacing, grammar, and consistency.

Reviewer 2

"Line 55: Consider replacing "parts" with "aspects" for improved clarity."

We agree and replaced "parts" with "aspects".

"Methods Section: Please include a dedicated METHODS section with an appropriate subheading placed after the Introduction"

We thank the reviewer for this suggestion. We agree that a clearly structured Methods section is important for readability. However, the journal's manuscript format requires the Methods section to appear after the Results/Discussion rather than immediately after the Introduction. We therefore retained the section in the journal-required position, but revised and expanded it for clarity.

"Discussion – Conceptual Clarity:

It would be helpful to distinguish more explicitly between familiarity and comfort. Specifically:

How did the authors ensure that survey language allowed respondents to meaningfully differentiate between these constructs?

Additionally, please elaborate on whether comfort reflects a risk-averse/tolerance-based position or a more enduring, benefit-driven acceptance"

We agree and clarified this distinction in the Methods and Discussion. In our revised wording, familiarity is operationalized through self-rated AI knowledge and reported AI use frequency, whereas comfort refers to respondents' stated willingness to accept AI in specific clinical roles. We also explicitly note that the survey cannot fully disentangle risk tolerance from perceived benefit because both constructs were self-reported. This clarification is consistent with the questionnaire structure in the supplement, which separates AI knowledge/use items from comfort items.

"Patient Engagement:

Consider expanding on strategies to enhance patient buy-in. What are the critical elements necessary to foster trust, engagement, and adoption in this context?"

Thank you, we agree and expanded the Discussion accordingly.

"Survey Design Limitation:

One limitation is that the survey questions appear predominantly positively oriented. There were no items exploring potential concerns, such as whether AI may be too risky or could cause harm.

This response bias and orientation limitation should be mentioned. And, other limitation of generalizability of the results across broader MS populations"

We agree and added this as a limitation. The questionnaire indeed focused primarily on perceived potential, comfort, and preference for AI-supported care, with limited explicit probing of possible harms or perceived risks, as also visible in the supplementary survey instrument.

Moreover, we agree that generalizability may be limited across broader MS populations. We have modified the limitations to reflect this feedback.

Considering the additional journal requirements, we revised the Figures using the PLOS's NAAS tool and uploaded the revised Figures 1 and 3. Moreover, we amended the Financial Disclosure statement following the instructions and added a full list of Supporting Information legends after the references.

Thank you again for the helpful comments and review reports.

Yours sincerely,

Prof. Dr. Tjalf Ziemssen, MD. Dr. Hernan Inojosa, MD.

On behalf of all authors.
