# Supplementary material for: Exploring attitudes and acceptance of artificial intelligence in multiple sclerosis from the patient perspective
Source: PLOS Digit Health. 2026 Jul 1;5(7):e0001236. doi: 10.1371/journal.pdig.0001236 (PMC13322512; doi:10.1371/journal.pdig.0001236)
Supplement: Peer review history

# Peer review: Decision Letter 0

**DOI:** 10.1371/journal.pdig.0001236.r001

**Authors:** Po-Chih Kuo

30 Mar 2026

PDIG-D-26-00145

Exploring Attitudes and Acceptance of Artificial Intelligence in Multiple Sclerosis from the Patient Perspective

PLOS Digital Health

Dear Dr. Ziemssen,

Thank you for submitting your manuscript to PLOS Digital Health. After careful consideration, we feel that it has merit but does not fully meet PLOS Digital Health's publication criteria as it currently stands. Therefore, we invite you to submit a revised version of the manuscript that addresses the points raised during the review process.

Please submit your revised manuscript by May 29 2026 11:59PM. If you will need more time than this to complete your revisions, please reply to this message or contact the journal office at digitalhealth@plos.org. When you're ready to submit your revision, log on to https://www.editorialmanager.com/pdig/ and select the 'Submissions Needing Revision' folder to locate your manuscript file.

Please include the following items when submitting your revised manuscript:

* A letter that responds to each point raised by the editor and reviewer(s). You should upload this letter as a separate file labeled 'Response to Reviewers'. This file does not need to include responses to any formatting updates and technical items listed in the 'Journal Requirements' section below.

* A marked-up copy of your manuscript that highlights changes made to the original version. You should upload this as a separate file labeled 'Revised Manuscript with Track Changes'.

* An unmarked version of your revised paper without tracked changes. You should upload this as a separate file labeled 'Manuscript'.

If you would like to make changes to your financial disclosure, competing interests statement, or data availability statement, please make these updates within the submission form at the time of resubmission. Guidelines for resubmitting your figure files are available below the reviewer comments at the end of this letter.

We look forward to receiving your revised manuscript.

Kind regards,

Po-Chih Kuo, Ph. D.

Section Editor

PLOS Digital Health

Po-Chih Kuo

Section Editor

PLOS Digital Health

Leo Anthony Celi

Editor-in-Chief

PLOS Digital Health

orcid.org/0000-0001-6712-6626

**Journal Requirements:**

1. Please amend your detailed Financial Disclosure statement. This is published with the article. It must therefore be completed in full sentences and contain the exact wording you wish to be published.

a. State the initials, alongside each funding source, of each author to receive each grant.

b. State what role the funders took in the study. If the funders had no role in your study, please state: “The funders had no role in study design, data collection and analysis, decision to publish, or preparation of the manuscript.”

c. If any authors received a salary from any of your funders, please state which authors and which funders.

2. We have noticed that you have uploaded Supporting Information files, but you have not included a list of legends. Please add a full list of legends for your Supporting Information files after the references list.

If the reviewer comments include a recommendation to cite specific previously published works, please review and evaluate these publications to determine whether they are relevant and should be cited. There is no requirement to cite these works unless the editor has indicated otherwise.

**Additional Editor Comments (if provided):**

[Note: HTML markup is below. Please do not edit.]

**Reviewers' Comments:**

Reviewer's Responses to Questions

**Comments to the Author**

1. Does this manuscript meet PLOS Digital Health’s publication criteria? Is the manuscript technically sound, and do the data support the conclusions? The manuscript must describe methodologically and ethically rigorous research with conclusions that are appropriately drawn based on the data presented.

Reviewer #1: Partly

Reviewer #2: Yes

**********

2. Has the statistical analysis been performed appropriately and rigorously?

Reviewer #1: I don't know

Reviewer #2: Yes

**********

3. Have the authors made all data underlying the findings in their manuscript fully available (please refer to the Data Availability Statement at the start of the manuscript PDF file)?

The PLOS Data policy requires authors to make all data underlying the findings described in their manuscript fully available without restriction, with rare exception. The data should be provided as part of the manuscript or its supporting information, or deposited to a public repository. For example, in addition to summary statistics, the data points behind means, medians and variance measures should be available. If there are restrictions on publicly sharing data—e.g. participant privacy or use of data from a third party—those must be specified.

Reviewer #1: Yes

Reviewer #2: Yes

**********

4. Is the manuscript presented in an intelligible fashion and written in standard English?

PLOS Digital Health does not copyedit accepted manuscripts, so the language in submitted articles must be clear, correct, and unambiguous. Any typographical or grammatical errors should be corrected at revision, so please note any specific errors here.

Reviewer #1: Yes

Reviewer #2: Yes

**********

5. Review Comments to the Author

Please use the space provided to explain your answers to the questions above. You may also include additional comments for the author, including concerns about dual publication, research ethics, or publication ethics. (Please upload your review as an attachment if it exceeds 20,000 characters)

Reviewer #1: This manuscript presents a cross-sectional, web-based survey of people with multiple sclerosis (pwMS) in Germany (N=241) assessing attitudes toward and acceptance of AI across eight MS-care domains. The topic is timely and relevant to digital neurology and implementation research. The manuscript is generally clear, and the “responsibility gradient” framing is conceptually useful. The supplementary material improves transparency by providing the full questionnaire, clearer operational definitions, and regression outputs.

Major concerns

Ma-1) Recruitment strategy and selection bias

Concern: Recruitment through two academic MS centers and multiple digital channels (social media/newsletter/podcast/website) strongly selects for digitally engaged respondents and may create systematic differences by recruitment pathway. The observed East/West differences in AI acceptance may reflect recruitment mix and center catchment rather than true regional variation.

Requested revision:

•Report recruitment sources quantitatively (e.g., center A vs center B; clinic vs online; specific channels if available).

•Compare key baseline characteristics and AI familiarity/usage by recruitment source.

•Re-estimate the “region” association adjusting for recruitment channel/center, or provide sensitivity analyses stratified by recruitment source.

•Temper interpretation of East/West differences unless robustness is demonstrated.

Ma-2) Missing data and denominators need systematic reporting and consistent handling

Concern: The supplementary material indicates substantial missingness for some variables (e.g., region responses not available for all participants). Variable denominators differ across analyses; however, the main manuscript does not provide a clear missingness overview or a consistent analytic approach.

Requested revision:

•Add a missingness table (per variable and per key item/domain) and explicitly state how missing data were handled in each model.

•Ensure all tables/figures clearly state denominators (N) for each item/domain and for subgroup comparisons.

Ma-3) Outcome processing and dichotomization risk information loss

Concern: Several predictors are dichotomized (e.g., median splits for age/disease duration; categorical collapses for Likert outcomes). While the supplementary clarifies how thresholds were chosen, these decisions still reduce information and may attenuate or distort associations.

Requested revision:

•Consider continuous modeling for age and disease duration (with non-linearity checks if needed).

•Provide sensitivity analyses using the full 5-level ordinal outcomes (or justify the collapsing empirically).

Ma-4) Reverse causality and self-selection

Concern: The observed association between frequent AI use and greater acceptance is plausible but cross-sectional. Reverse causality is equally plausible (those favorable to AI are more likely to use it). Survey participation itself may also be correlated with positive attitudes toward digital tools.

Requested revision:

•Replace causal language (“driven by,” “key lever”) with associative phrasing.

•Expand limitations to explicitly discuss reverse causality and self-selection.

Ma-5) Internal numerical inconsistency in Table 2

Concern: Table 2 contains at least one implausible estimate: “Health-related AI use” shows B=0.070 with a 95% CI 0.251 to 0.391, which cannot be correct (CI does not include the point estimate and is far from it). This undermines confidence in the regression output and needs correction.

Requested revision:

•Audit all coefficients, confidence intervals, and denominators across manuscript and supplement; ensure tables reproduce the model outputs exactly.

Ma-6) Survey reporting completeness: consider using the CROSS checklist

Concern: Several reporting gaps (recruitment sources/denominators, missingness, questionnaire development and validation steps) are common in survey research and can be addressed systematically.

Requested revision:

•I recommend reporting the study in line with the CROSS (Consensus-Based Checklist for Reporting of Survey Studies), either by adding a completed checklist as supplementary material and/or explicitly mapping key items (sampling frame, recruitment, response rates, instrument development, missing data handling) in the Methods and Supplement. This would improve transparency and facilitate reproducibility.

Minor concerns

1.Consider adjusting (or at least describing) potential center effects, given recruitment from two academic centers.

2.Minor language/formatting edits (typos, spacing, consistent terminology) to improve readability.

Reviewer #2: Line 55: Consider replacing “parts” with “aspects” for improved clarity.

Methods Section: Please include a dedicated METHODS section with an appropriate subheading placed after the Introduction

Discussion – Conceptual Clarity:

It would be helpful to distinguish more explicitly between familiarity and comfort. Specifically:

How did the authors ensure that survey language allowed respondents to meaningfully differentiate between these constructs?

Additionally, please elaborate on whether comfort reflects a risk-averse/tolerance-based position or a more enduring, benefit-driven acceptance.

Patient Engagement:

Consider expanding on strategies to enhance patient buy-in. What are the critical elements necessary to foster trust, engagement, and adoption in this context?

Survey Design Limitation:

One limitation is that the survey questions appear predominantly positively oriented. There were no items exploring potential concerns, such as whether AI may be too risky or could cause harm.

This response bias and orientation limitation should be mentioned. And, other limitation of generalizability of the results across broader MS populations.

**********

6. PLOS authors have the option to publish the peer review history of their article (what does this mean?). If published, this will include your full peer review and any attached files.

**Do you want your identity to be public for this peer review?**  If you choose “no”, your identity will remain anonymous but your review may still be made public.

For information about this choice, including consent withdrawal, please see our Privacy Policy.

Reviewer #1: **Yes:** Simone Salemme

Reviewer #2: **Yes:** Fawad Khan

**********

[NOTE: If reviewer comments were submitted as an attachment file, they will be attached to this email and accessible via the submission site. Please log into your account, locate the manuscript record, and check for the action link "View Attachments". If this link does not appear, there are no attachment files.]

**Figure resubmission:**

While revising your submission, we strongly recommend that you use PLOS’s NAAS tool (https://ngplosjournals.pagemajik.ai/artanalysis) to test your figure files. NAAS can convert your figure files to the TIFF file type and meet basic requirements (such as print size, resolution), or provide you with a report on issues that do not meet our requirements and that NAAS cannot fix.

After uploading your figures to PLOS’s NAAS tool - https://ngplosjournals.pagemajik.ai/artanalysis, NAAS will process the files provided and display the results in the "Uploaded Files" section of the page as the processing is complete. If the uploaded figures meet our requirements (or NAAS is able to fix the files to meet our requirements), the figure will be marked as "fixed" above. If NAAS is unable to fix the files, a red "failed" label will appear above. When NAAS has confirmed that the figure files meet our requirements, please download the file via the download option, and include these NAAS processed figure files when submitting your revised manuscript.

**Reproducibility:**

To enhance the reproducibility of your results, we recommend that authors of applicable studies deposit laboratory protocols in protocols.io, where a protocol can be assigned its own identifier (DOI) such that it can be cited independently in the future. Additionally, PLOS ONE offers an option to publish peer-reviewed clinical study protocols. Read more information on sharing protocols at https://plos.org/protocols?utm_medium=editorial-email&utm_source=authorletters&utm_campaign=protocols

# Author comment: Author response to Decision Letter 1

**DOI:** 10.1371/journal.pdig.0001236.r002

15 Apr 2026

Attachment Submitted filename: Response to Reviewers_PLOS_[PDIG-D-26-00145].pdf

# Peer review: Decision Letter 1

**DOI:** 10.1371/journal.pdig.0001236.r003

**Authors:** Po-Chih Kuo, Po-Chih Kuo

4 Jun 2026

Exploring Attitudes and Acceptance of Artificial Intelligence in Multiple Sclerosis from the Patient Perspective

PDIG-D-26-00145R1

Dear Prof. Ziemssen,

We are pleased to inform you that your manuscript 'Exploring Attitudes and Acceptance of Artificial Intelligence in Multiple Sclerosis from the Patient Perspective' has been provisionally accepted for publication in PLOS Digital Health.

Before your manuscript can be formally accepted you will need to complete some formatting changes, which you will receive in a follow-up email from a member of our team.

Please note that your manuscript will not be scheduled for publication until you have made the required changes, so a swift response is appreciated.

IMPORTANT: The editorial review process is now complete. PLOS will only permit corrections to spelling, formatting or significant scientific errors from this point onwards. Requests for major changes, or any which affect the scientific understanding of your work, will cause delays to the publication date of your manuscript.

If your institution or institutions have a press office, please notify them about your upcoming paper to help maximize its impact. If they'll be preparing press materials, please inform our press team as soon as possible -- no later than 48 hours after receiving the formal acceptance. Your manuscript will remain under strict press embargo until 2 pm Eastern Time on the date of publication. For more information, please contact digitalhealth@plos.org.

Thank you again for supporting Open Access publishing; we are looking forward to publishing your work in PLOS Digital Health.

Best regards,

Po-Chih Kuo, Ph. D.

Section Editor

PLOS Digital Health

***********************************************************

**Additional Editor Comments (if provided):**

**Reviewer Comments (if any, and for reference):**

Reviewer's Responses to Questions

**Comments to the Author**

1. If the authors have adequately addressed your comments raised in a previous round of review and you feel that this manuscript is now acceptable for publication, you may indicate that here to bypass the “Comments to the Author” section, enter your conflict of interest statement in the “Confidential to Editor” section, and submit your "Accept" recommendation.

Reviewer #1: All comments have been addressed

Reviewer #2: All comments have been addressed

**********

2. Does this manuscript meet PLOS Digital Health’s publication criteria? Is the manuscript technically sound, and do the data support the conclusions? The manuscript must describe methodologically and ethically rigorous research with conclusions that are appropriately drawn based on the data presented.

Reviewer #1: (No Response)

Reviewer #2: Yes

**********

3. Has the statistical analysis been performed appropriately and rigorously?

Reviewer #1: (No Response)

Reviewer #2: N/A

**********

4. Have the authors made all data underlying the findings in their manuscript fully available (please refer to the Data Availability Statement at the start of the manuscript PDF file)?

The PLOS Data policy requires authors to make all data underlying the findings described in their manuscript fully available without restriction, with rare exception. The data should be provided as part of the manuscript or its supporting information, or deposited to a public repository. For example, in addition to summary statistics, the data points behind means, medians and variance measures should be available. If there are restrictions on publicly sharing data—e.g. participant privacy or use of data from a third party—those must be specified.

Reviewer #1: (No Response)

Reviewer #2: Yes

**********

5. Is the manuscript presented in an intelligible fashion and written in standard English?<br/><br/>PLOS Digital Health does not copyedit accepted manuscripts, so the language in submitted articles must be clear, correct, and unambiguous. Any typographical or grammatical errors should be corrected at revision, so please note any specific errors here.

Reviewer #1: (No Response)

Reviewer #2: Yes

**********

6. Review Comments to the Author<br/><br/>Please use the space provided to explain your answers to the questions above. You may also include additional comments for the author, including concerns about dual publication, research ethics, or publication ethics. (Please upload your review as an attachment if it exceeds 20,000 characters)

Reviewer #1: (No Response)

Reviewer #2: No further comments. Thank you for making these improvements.

**********

7. PLOS authors have the option to publish the peer review history of their article (what does this mean?). If published, this will include your full peer review and any attached files.

**Do you want your identity to be public for this peer review?**  If you choose “no”, your identity will remain anonymous but your review may still be made public.

For information about this choice, including consent withdrawal, please see our Privacy Policy.

Reviewer #1: **Yes:** Simone Salemme

Reviewer #2: **Yes:** Fawad A. Khan

**********
